# Making the “Birthing-Friendly” hospital designation better

**DOI:** 10.1093/haschl/qxaf170

**Published:** 2025-08-26

**Authors:** Amanda Bonheur, Kortney Floyd James, Megan Andrew

**Affiliations:** RAND Corporation, Economics, Sociology & Statistics Department, Washington, D.C. 22202, United States; RAND Corporation, Behavioral & Policy Sciences Department, Santa Monica, CA 90401, United States; RAND Corporation, Economics, Sociology & Statistics Department, Pittsburgh, PA 15213, United States

**Keywords:** Birthing-Friendly designation, maternal health disparities, health equity, health care policy

## Abstract

The “Birthing-Friendly” designation, intended to guide birthing individuals toward quality hospitals, has become widespread. However, our analysis of hospital data finds that the Birthing-Friendly designation does not differentiate hospitals based on meaningful quality measures. Our analysis shows that while Birthing-Friendly hospitals are larger and engage in quality improvement efforts, they do not consistently outperform non-designated hospitals on core maternal health metrics such as early elective delivery rates or births-to-staff ratios. The designation likely reflects a hospital's capacity to adopt basic quality improvement programming structures more than its ability to provide consistent, high-quality maternal care. To address this, we propose a more robust measure that includes clinical outcomes, patient experiences, and equity metrics, particularly for marginalized groups like Black and Indigenous birthing people.

## Introduction

The United States is experiencing a maternal health crisis, with birthing parents dying from pregnancy-related causes at higher rates than any other high-income country.^1^ The risk is over 200% higher for Black and Native American birthing people.^2^ Experts suggest that as much as 84% of pregnancy-related deaths are preventable.^3^ With nearly all US births taking place in hospitals,^4^ health care and hospital systems play a critical role in addressing the quality of care throughout the perinatal period.

The Centers for Medicare & Medicaid Services (CMS) introduced the “Birthing-Friendly” hospital designation in 2022 to help individuals select a quality hospital to safely give birth. While the designation is the first step in CMS’ larger plan to measure and disseminate information on the quality of birthing care in US hospitals, it falls far short of conveying meaningful insights into the actual quality of perinatal care and will likely have limited impact on the US maternal health crisis as a result.

## What is the Birthing-Friendly designation?

In June 2022, the Biden Administration released the White House Blueprint for Addressing the Maternal Health Crisis.^5^ As part of this blueprint, they proposed a Birthing-Friendly designation for hospitals that (1) participate in a national or state perinatal quality improvement collaborative program and (2) implement evidence-based best practices to improve maternal health. Hospitals that self-attest to meeting both are granted the designation.^6^

CMS began collecting data on hospital participation in perinatal quality improvement collaborative programs and the implementation of evidence-based best practices in 2022 through its Hospital Inpatient Quality Reporting (IQR) Program.^7^ This program, required for all US hospitals providing acute care and receiving Medicare payments for in-patient care,^8^ covers over 88% of US hospitals and provides comprehensive data on quality maternal care.^9^ In November 2023, the Birthing-Friendly designation was added to the CMS Care Compare tool to help birthing individuals identify hospitals offering quality labor and delivery care.^10^

Research on how patients choose hospitals for maternity care shows mixed findings. While many prioritize provider relationships, location, and insurance over formal quality ratings,^11^ others—particularly those with prior birth experience or living in metropolitan areas—do report considering hospital reputation and perceived quality.^12^ These findings suggest that although hospital quality matters to some patients, current reporting formats may not be accessible, trusted, or actionable enough to consistently inform decision-making.

**Figure 1.**
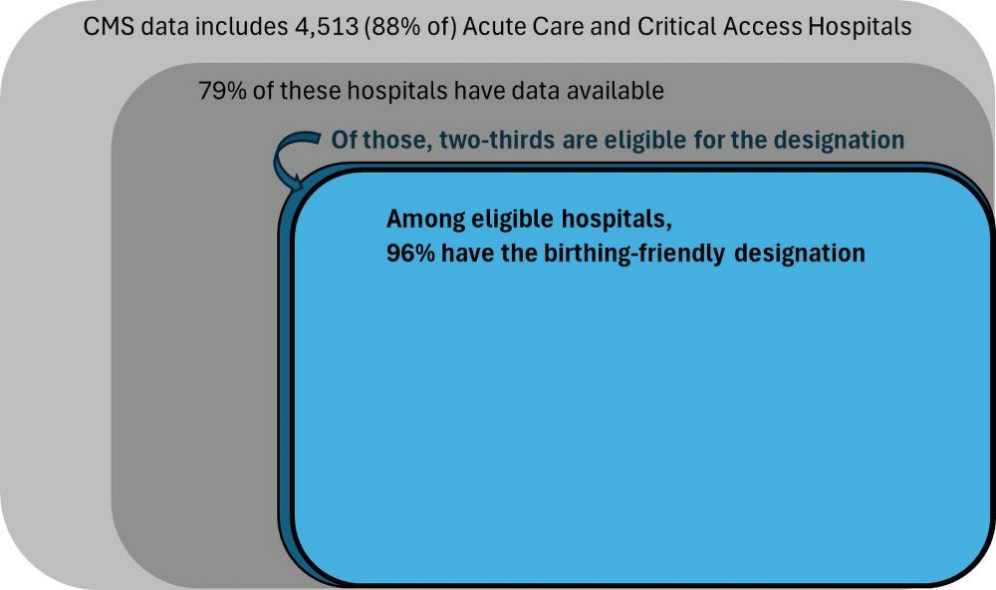
96% of eligible US hospitals receive the Birthing-Friendly designation. Source: Authors’ representation of 2023 CMS data. Notes: Eligible hospitals are those with a labor and delivery or obstetric unit.

Based on our analysis of CMS data, nearly all eligible hospitals (96%) receive the Birthing-Friendly designation (Figure 1). This pattern is especially striking when compared to a similar designation in the United Kingdom. Maternal mortality rates are lower in the United Kingdom than in the United States,^1^ yet a 2023 Care Quality Commission report found that 67% of birthing units in the United Kingdom were rated as “requires improvement” or “inadequate” for safety,^13^ meaning that only 33% received an adequate rating. This contrast highlights how other similar designations might better reflect quality perinatal care during a hospital birth, compared to the CMS designation for US hospitals.

## Birthing-Friendly hospitals do not outperform other hospitals on standard quality measures

While the designation signals participation in quality improvement (QI) efforts, it does not appear to correlate with higher performance on standard measures of quality maternal care. Using 2022 data from the CMS IQR and the American Hospital Association's (AHA) annual survey, we compared Birthing-Friendly and non-Birthing-Friendly hospitals across quality measures. Hospitals with the designation were generally larger, with a median of 965 births in 2022, compared to 351 births in non-designated hospitals. They also had more obstetric care beds and staff and were more likely to be in metropolitan areas. However, 89% of rural hospitals were also designated Birthing-Friendly, underscoring how widespread the classification has become.

**Figure 2.**
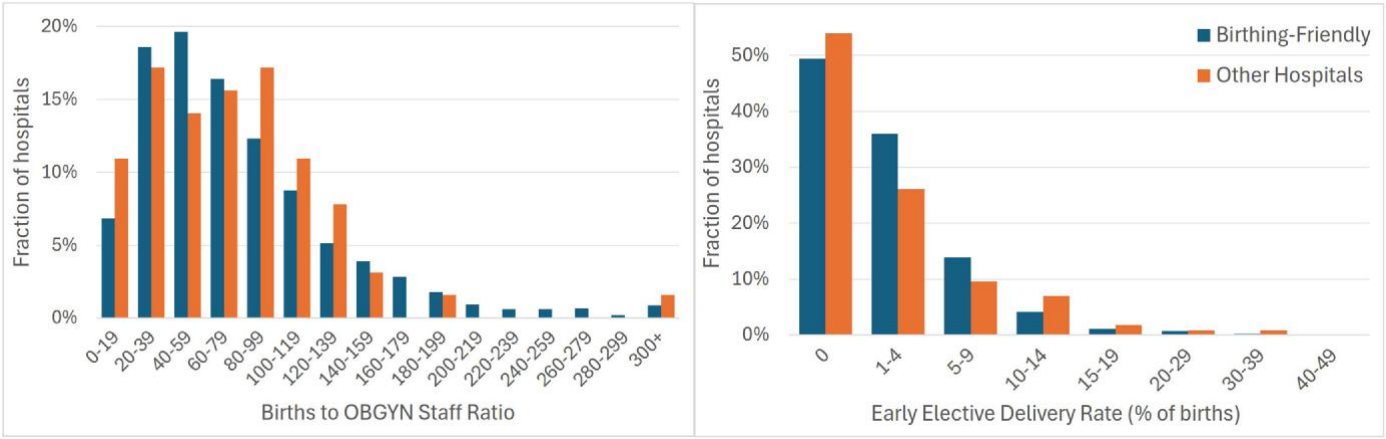
Birthing-Friendly and other hospitals have similar births-to-obstetrics-and-gynecology-staff ratios and early elective delivery rates. Source: Authors’ representation of 2022 CMS and 2022 AHA data. Notes: Other hospitals refer to those who do not have the Birthing-Friendly designation but are eligible for it because they have a labor and delivery or obstetric unit. Births-to-OBGYN-Staff ratios are calculated for hospitals that have data available for both number of births and total privileged OBGYN staff and had at least 1 birth during that year. There are *N* = 1518 Birthing-Friendly hospitals and *N* = 64 Other hospitals with births:OBGYN staff ratios. Elective delivery rates are directly collected by CMS and represent the percentage of patients whose deliveries were scheduled 1-2 weeks early without medical necessity; lower percentages are considered better. There are *N* = 2103 Birthing-Friendly hospitals and *N* = 115 Other hospitals with a non-missing elective delivery rate.

Despite these differences in infrastructure, designated hospitals did not significantly outperform their non-designated counterparts on core measures of quality. Distributions of early elective delivery rates and births-to-obstetrics-and-gynecology-staff ratios were largely comparable across hospitals that did and did not receive the designation (Figure 2). Early elective delivery rates are a key measure of maternal care^14^ and better staffing ratios improve birthing outcomes.^15,16^ Neither measure is statistically significantly different between Birthing-Friendly and other hospitals. Births-to-OBGYN-staff ratios among Birthing-Friendly hospitals (*M* = 79.9, SD = 63.5) are similar to those among other hospitals (*M* = 74.2, SD = 53.5), *t*(1580) = −0.71, *P* = 0.48 (The distributions of births-to-OBGYN-staff-ratios are also statistically the same between Birthing-Friendly and other eligible hospitals according to the Kolmogorov-Smirnov test (*P* = 0.928). Early elective delivery rates among Birthing-Friendly hospitals (*M* = 2.46, SD = 4.08) are statistically equal to those among other hospitals (*M* = 2.92, SD = 5.56), *t*(2216) = 1.15, *P* = 0.25. It is possible that there are small differences we are unable to detect due to the smaller sample of non-Birthing-Friendly hospitals. At the same time, prior research shows that Birthing-Friendly hospitals in the United States do not consistently meet benchmarks on other key maternal care outcomes, like cesarean delivery rates.^17^ These findings suggest that among measures we are able to evaluate, the designation does not meaningfully differentiate hospitals in terms of quality of provided care.

## The designation might reflect hospital capacity for programming structure, but not higher quality maternal care

**Figure 3.**
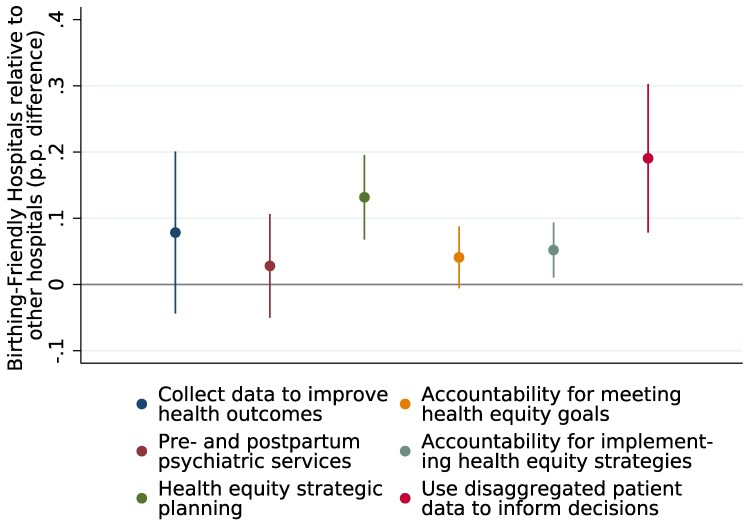
Birthing-Friendly hospitals are more likely to implement general quality improvement measures at the hospital level. Source: Authors’ analysis of 2022 AHA data, with hospitals classified using 2022 CMS data. Notes: Graph shows regression coefficients of the differences between Birthing-Friendly and other hospitals that are eligible for the Birthing-Friendly designation, controlling for the number of births, type of hospital, and urbanization of the hospital's location.

We also examined the implementation of QI measures using AHA and CMS data. Designated hospitals were more likely to engage in QI efforts at the hospital level, including strategic planning for health equity, establishing accountability structures, and using disaggregated patient data to inform QI initiatives, even when controlling for hospital characteristics (Figure 3). However, the designation does not correlate with better outcomes on key maternal care measures (Figure 2).

Together, these findings suggest that the Birthing-Friendly designation reflects a hospital's capacity for putting QI programming structure in place more than it indicates consistent implementation of quality maternal care that lead to improved outcomes for birthing patients. This aligns with findings from implementation science, which show significant variability in the implementation of similar QI programs across hospitals.^18^ In our own ongoing research^19^ on state perinatal quality care collaborative safety bundles, nurse managers in labor and delivery units consistently report barriers to consistent QI implementation, such as limited financial resources and competing priorities for non-maternal QI efforts. For instance, nurse managers shared challenges in purchasing basic equipment, such as scales to measure maternal blood loss, for maternal hemorrhage safety bundles. Given these gaps in consistent implementation, the Birthing-Friendly designation, while promising, does not fully address the needs of patients seeking reliable, high-quality maternal care.

While our analysis suggests that Birthing-Friendly hospitals are more likely to implement QI structures, the relationship between these activities and actual improvements in maternal outcomes is far from straightforward. Research on perinatal safety bundles and collaborative models shows that QI efforts can reduce severe maternal morbidity, particularly for conditions like hemorrhage and hypertension, but only when implemented consistently and supported by adequate resources, strong leadership, and data systems to monitor performance, and accountability mechanisms.^20,21^

In other words, the presence of QI structures may reflect that a hospital is organizationally prepared to pursue improvement, but this does not necessarily mean that those efforts are translating into better care at the bedside. This distinction is especially important in the context of a public-facing designation. Without mechanisms to assess fidelity of implementation or actual clinical outcomes, the Birthing-Friendly label may reward hospitals for participation rather than performance—and in doing so, obscure meaningful differences in the quality of maternal care.

## Toward a more nuanced measure of quality maternity care

Improving the Birthing-Friendly designation, or replacing it with a more rigorous classification, requires rethinking how quality is defined and measured in maternity care. CMS has indicated plans to propose a more robust set of criteria for the Birthing-Friendly designation in the future,^17,22^ and we echo the calls from fellow health policy professionals for urgent action in this regard, especially in light of our analysis.^14^ A more meaningful approach would incorporate not only structural indicators, such as hospital size and staffing, but also patient experiences, clinical outcomes, and performance on equity measures. This is particularly important for addressing disparities in maternal health outcomes, especially among Black and Indigenous communities, who experience poorer outcomes due to systemic marginalization and poor-quality care. Current quality indicators offer limited practical value to these populations, and it is critical that any revised designation serves their needs and reflects their lived experiences.

A revised designation could include indicators such as rates of cesarean delivery among low-risk nulliparous patients, postpartum readmission, severe maternal morbidity rates, and participation in maternal mortality or morbidity reviews. CMS’ plan to include 2 new clinical quality measures, cesarean births and severe obstetric complications, in the IQR reporting requirement for hospitals in fiscal year 2026, shows progress.^22^ However, CMS should work quickly to incorporate these measures into its public-facing Birthing-Friendly designation.^17^ Incorporating patient-reported experience measures (PREM), such as those adapted from the Mothers on Respect Index^23^ or Obstetric Racism ©,^24^ would also better reflect whether care is perceived as respectful, culturally aligned, and trauma-informed. These measures are not yet collected systematically across maternity hospitals, and widespread implementation would require federal leadership, standardized tools, integration into existing quality reporting infrastructure, and technical assistance for hospitals—particularly those with limited capacity. Hospitals could also be assessed on their integration of midwifery, availability of doula or community-based services, and the extent to which they provide language access and equitable care to diverse patient populations. Importantly, these indicators should be disaggregated by race, ethnicity, insurance status, and preferred language to ensure transparency and accountability in serving marginalized communities.

Equally important, the designation should carry material significance. Currently, the Birthing-Friendly label functions more as a passive indicator than a driver of change. To incentivize real progress, CMS could align the designation with reimbursement mechanisms, such as value-based payment models, public reporting, or differential incentive structures for hospitals demonstrating year-over-year improvement. Rather than a binary classification, a tiered or leveled designation system, similar to Magnet^25^ or Baby-Friendly Hospital^26^ status, could recognize progress over time and encourage hospitals to strengthen their maternal care infrastructure in ways that meaningfully advance equity.

Broader evidence from hospital quality reporting underscores both the value and limitations of these strategies. Transparency (ie, making performance data public) is an important step, but on its own has not consistently improved clinical outcomes or influenced patient decision-making.^27^ Without meaningful financial incentives or regulatory accountability, reporting often becomes performative rather than transformative. On the other hand, tying quality metrics to payment, as seen in Medicare's Hospital Readmissions Reduction Program, can prompt improvement but also introduces real risks, particularly for safety-net hospitals serving structurally marginalized communities.^28^ These facilities may be penalized for outcomes driven by factors beyond their control, including structural inequities in housing, transportation, and access to prenatal care. Maternity care is especially vulnerable to these dynamics, given the stark racial disparities in outcomes and the uneven distribution of clinical resources. For any future pay-for-performance model linked to the Birthing-Friendly designation, equity must be foundational, not an afterthought. This means incorporating risk adjustment, establishing equity-focused benchmarks, and providing targeted support to under-resourced hospitals working to improve care.

Given the scope of the maternal health crisis in the United States, particularly among Black and Indigenous birthing people, it is no longer sufficient to emphasize participation in QI collaboratives without evaluating the substance and outcomes of those efforts. A redesigned Birthing-Friendly designation should reflect measurable improvements in clinical care and patient experience to provide actionable guidance for families, clinicians, researchers, and policymakers. This would ensure that patients, especially those from marginalized communities, have meaningful information to make informed decisions about where to find safe, respectful, and high-quality maternal care.

## Conclusion

The Birthing-Friendly designation is an important starting point in recognizing the role of hospital systems in improving maternal care and health, but it remains an incomplete measure. In the context of the persistent and racialized maternal health crisis, the designation must do more than signal participation in improvement efforts—it must reflect real differences in care. By aligning the designation with meaningful clinical outcomes, equity metrics, and patient experiences, and tying it to CMS accountability mechanisms, we can move toward measuring quality maternal care. There is an opportunity now for researchers, policymakers, and health systems to collaborate on a more rigorous, equitable standard—one that truly helps birthing people identify where they are most likely to receive safe, respectful, and high-quality care.

## Supplementary Material

qxaf170_Supplementary_Data
